# Establishment of Self-Renewable GM-CSF-Dependent Immature Macrophages *In Vitro* from Murine Bone Marrow

**DOI:** 10.1371/journal.pone.0076943

**Published:** 2013-10-04

**Authors:** Sachiko Ito, Yuriko Tanaka, Naomi Nishio, Suganya Thanasegaran, Ken-Ichi Isobe

**Affiliations:** Department of Immunology, Nagoya University Graduate School of Medicine, Nagoya, Aichi, Japan; Rutgers - New Jersey Medical School, United States of America

## Abstract

Macrophages play a key role in the innate immune system. Macrophages are thought to originate from hematopoietic precursors or the yolk sac. Here, we describe the *in vitro* establishment of self-renewable GM-CSF-dependent immature macrophages (GM-IMs) from murine bone marrow (BM). GM-IMs grow continuously *in vitro* in conditioned medium containing GM-CSF. The immunophenotype of GM-IMs is F4/80^high^ CD11b^high^ CD11c^low^ Ly6C^low^. By comparing gene expression in GM-IMs and BM dendritic cells, we found that GM-IMs expressed lower levels of chemokines, cytokines and their receptors. GM-IMs are round in shape, attach loosely to non-coated culture dishes and have a marked phagocytic capacity. These results indicate that GM-IMs are macrophage precursor cells. Following stimulation with LPS, monocyte-like GM-IMs converted to flat macrophage-like cells that tightly adhered to non-coated culture dishes and produced pro-inflammatory cytokines TNFα, IL-6 and IL-1β. These results indicated that GM-IMs differentiated to M1 pro-inflammatory macrophages. This was confirmed by stimulation of GM-IMs with IFNγ, an inducer of M1 markers. GM-IMs showed enhanced expression of M2 macrophage markers such as *Arg1* and *Retnla* following stimulation by Th2 cytokines IL-4 and IL-13. When GM-IMs were injected into mice at sites of wounding, wound repair was enhanced. These results indicate that GM-IMs can differentiate to M2 macrophages. When GM-IMs were injected into clodronate-treated mice, they induced resident macrophage proliferation by producing M-CSF. In conclusion we have established self-renewable GM-CSF-dependent immature macrophages *in vitro* from murine BM, which differentiate to M1 or M2 macrophages.

## Introduction

Macrophages and dendritic cells (DCs) are present in all tissues and are crucial for immune and inflammatory responses. They belong to a network of cells that has been termed the mononuclear phagocyte system (MPS) [1]. The MPS originates from bone marrow (BM) hematopoietic stem cells. Macrophage precursors are released into the circulation as monocytes, and within a few days they seed tissues throughout the body, including the spleen, which serves as a storage reservoir for immature monocytes [2,3]. However, the origin and lineage of these cells are still controversial. In adults, hematopoietic stem cells (HSCs) give rise to most macrophages and they are replaced continually by macrophage and DC precursors [4,5]. In adult mammals, HSCs also give rise to circulating monocytes. All resident macrophages in tissues may be derived from circulating monocytes [2]. However, this model of the development of the MPS has been challenged recently. In vertebrate embryos, two different types of hematopoietic cells can give rise to macrophages [6,7]. In mice, on embryonic day 8 (E8), the yolk sac (YS) gives rise to macrophages [8]. Then, definitive HSCs emerge from the mouse hematogenic endothelium of the aorto-gonadal-mesonephros region at E10.5 [9,10]. At later times, the fetal liver is the source of definitive hematopoiesis that generates circulating monocytes during embryogenesis. Coincident with the postnatal formation of bone, fetal liver hematopoiesis declines and is replaced by BM hematopoiesis. In classical experiments, Furth et al. showed the dual origin of tissue macrophages by labeling monocytes from BM with [^3^H]thymidine. They showed that 55% of spleen macrophages were supplied by monocyte influx and 45% by local production [11]. Recently, it was shown that YS-derived F4/80 bright macrophages are present in several tissues of adult mice and are distinct from HSC progeny [12].

Functional studies have shown that macrophages originate in BM as monocytes. Circulating Ly6c^high^ monocytes enter the inflamed tissues and become M1 macrophages (activated macrophages or inflammatory macrophages) and resident Ly6c^low^ monocytes become M2 macrophages (resident macrophages or activated macrophages) [3,13]. These two states are defined by responses to interferon-γ (IFN-γ) and activation of Toll-like receptors (TLRs), and to interleukin-4 (IL-4) and IL-13, respectively [14,15]. Here, we established GM-CSF-dependent self-renewable immature macrophages (GM-IMs) by continued culture in GM-CSF-conditioned medium (CM). The cells constitute a single population and have continued to proliferate over the long-term. GM-IMs could differentiate to M1 or M2 macrophage subpopulations.

## Materials and Methods

### Mice

Two-month-old C57BL/6N mice were purchased from SLC Japan. EGFP-C57BL/6 mice (C14-Y01-FM131Osb) carrying pCAG-EGFP (CAGpromoter-EGFP) were purchased from Riken with the permission of Dr Okabe [16]. These mice were maintained in the Animal Research Facility at the Nagoya University Graduate School of Medicine under specific pathogen-free conditions and used according to institutional guidelines. This study was carried out in strict accordance with the recommendations of the Regulations on Animal Experimentation at Nagoya University. The Animal Care and Use Committee of Nagoya University Graduate School of Medicine approved the protocol. All experiments were performed under anesthesia and were designed to minimize suffering. Mice were humanely sacrificed prior to tissue collection.

### Cell culture

BM cells (3 x 10^6^) from C57BL/6N mice were cultured on 100 mm diameter non-coated plastic culture dishes using RPMI-1640 (Sigma) supplemented with penicillin, streptomycin (Invitrogen), 50 µM 2-mercaptoethanol, 10% heat-inactivated FBS (Gibco) and 10% medium conditioned by murine GM-CSF-producing Chinese hamster ovary (CHO) cells (GM-CSF-CM), a gift from Dr. T. Sudo, Toray Silicon, Tokyo, Japan. At day ten, non-adherent cells were collected as BMDC and attached cells were collected as BM macrophages (BMMφ). GM-IMs were continuously cultured with 10% GM-CSF-CM. To isolate peritoneal macrophages, mice were injected intraperitoneally with one mL of 3% (wt/vol) thioglycollate medium (Sigma). Three days later, cells were harvested from the peritoneal cavities by lavage with ice-cold PBS. RAW264.7 cells were obtained from RIKEN. Cells were cultured in DMEM (Sigma) with 10% heat-inactivated FBS. For growth curves, GM-IMs were sub-cultured every three or four days and the number of GM-IMs was measured by trypan blue dye exclusion using a Burker-Turk cell count chamber and calculated total cell number. Phase contrast photographs of cells were taken by using FSX100 bio imaging navigator (Olympus).

### Cell-cycle analysis

Cell cycle analyses were carried out using BrdU Flow kit according to the manufacturer’s instructions (BD Biosciences). In brief, before harvesting, cells were incubated with 0.3 mM BrdU in medium for one hr. Cells were collected, fixed and permeabilized with Cytofix/Cytoperm buffer, followed by incubation with DNase for one hr at 37°C. Samples were stained with FITC-labeled anti BrdU antibody and 7-AAD. Fluorescent signals were acquired using a FACSCanto (BD Biosciences), followed by data analysis using FlowJo software (TreeStar).

### Cytological analysis

Cells were collected and subjected to Cytospin (Thermo Scientific) processing. For morphological analysis, the cytospin preparations were fixed and stained with May-Grunwald-Giemsa. Briefly, air-dried cytospin slides were covered in May-Grunwald stain solution (Wako) for three min, after which one volume of PBS was added for three min. After rinsing in water, the slides were covered in 5% Giemsa stain solution (Wako) for 15 min. The slides were rinsed in water and air-dried. The slides were mounted with malinol (Muto Pure Chemicals). Photographs were taken by using FSX100 bio imaging navigator.

### Immunoblotting

Cells were lysed in 2 x SDS-sample buffer (62.5 mM Tris-HCl pH 6.8, 2% SDS, 20% glycerol, 5% 2-mercaptoethanol, and 0.025% bromophenol blue). Protein concentrations were quantified with the Lowry assay using the RC DC protein assay (Bio Rad). Then, 30 µg of total protein was resolved by SDS-PAGE and transferred to PVDF membranes (Millipore). Blotted membranes were incubated with 3% non-fat dry milk in PBS with 0.05% Tween-20 for 30 min to block nonspecific binding, and then the indicated antibodies were added for overnight incubation at 4°C. After one hr incubation at room temperature with HRP-linked anti-rabbit IgG (1:8000, GE Healthcare), protein bands were detected with ECL Prime (GE Healthcare) and analyzed with LumiVision Pro (Aishin). Antibodies to p16 (M-156; 1:500) were obtained from Santa Cruz. Anti-actin antibodies (1:4000) were from Sigma.

### Senescence-associated β- galactosidase (SA-β-gal) stain assay

Cells were cultured on 12-well plates and stained with a senescence detection kit according to the manufacturer’s instructions (BioVision). In brief, cells were washed with PBS and then treated with the Fixative Solution for 15 min. Cells were washed with PBS and stained with the Staining Solution Mixture overnight at 37°C. After staining, cells were covered with 70% glycerol.

### RT-PCR and quantitative RT-PCR

Total RNA was isolated from the cells using the RNeasy mini kit (Qiagen). RNA was quantified by NanoDrop (Thermo Scientific). cDNA was then synthesized from one µg total RNA with a high capacity cDNA reverse transcription kit according to the manufacturer’s instructions (Applied BioSystems). PCR was performed using Takara EX Taq (Takara Bio). Real time PCR was performed on the MX3000P QPCR System (Agilent) using the following program: ten sec at 95°C, followed by 40 cycles of five sec at 95°C, and 20 sec at 60°C. The reactions were carried out using 0.5 µL cDNA with SYBR premix EX TaqII (Takara Bio). Values were normalized to *Gapdh* mRNA. The primer sequences are shown in Table S1.

### Telomere measurement by quantitative PCR

Average telomere length was measured from total genomic DNA using a real-time PCR assay, as previously described [17]. In brief, genomic DNA was prepared using DNAzol (Invitrogen). PCR reactions were performed with SYBR premix EX Taq on a MX3000P QPCR System, using telomeric primers and mouse acidic ribosomal phosphoprotein PO (*36B4*) primers for the reference control gene and PCR settings as previously described [17]. Forward and reverse telomeric primers were as follows: 5′-CGG TTT GTT TGG GTT TGG GTT TGG GTT TGG GTT TGG GTT-3′, and 5′-GGC TTG CCT TAC CCT TAC CCT TAC CCT TAC CCT TAC CCT-3′, respectively. Forward and reverse primers for the *36B4* were as follows: 5′-ACT GGT CTA GGA CCC GAG AAG-3′ and 5′-TCA ATG GTG CCT CTG GAG ATT-3′, respectively. For each PCR reaction, a standard curve was made by serial dilutions of known amounts of DNA. The telomere signal was normalized to the signal from the *36B4* gene to generate average relative telomere lengths. Equal amounts of DNA (80 ng) were used for each reaction, with at least three replicates for each sample.

### Flow cytometry

Cells (1x10^6^) were suspended in 50 µL phosphate-buffered saline (PBS) supplemented with 1% FBS and stained for 20 min at 4°C with directly conjugated fluorescent antibodies (1:500). Whole blood was collected in tubes containing EDTA 2K. After staining, RBCs were lysed using a buffered ammonium chloride solution. Antibodies were as follows: fluorescein isothiocyanate (FITC)-conjugated anti-Gr1 was from BD Biosciences; anti-H-2d anti-CD86 was from eBioscience; phycoerythrin (PE)-conjugated anti-CD11b, anti-c-kit, and anti-CD34 were from BD Biosciences; anti-CD115 was from eBiosceince; allophycocyanin (APC)-conjugated anti-F4/80, anti-Sca-I were from eBioscience; eFluor450- conjugated anti-CD11b was from eBioscience; PE-Cyanine7-conjugated anti-CD11c was from eBioscience; PerCP-Cy5.5-conjugated anti-Ly6c was from eBiosceince. Stained cells were analyzed with the FACSCanto flow cytometer using FACS Diva software (BD Biosciences). For each sample, 10,000 cells were collected. All data were analyzed with FlowJo software.

### ELISA

The level of cytokine production in culture medium was measured by mouse Quantikine ELISA kits specific for IL-6, TNFα, IL-1β and IL-10 according to the manufacturer’s instructions (R & D systems).

### Phagocytosis

FITC-E*. coli* particles were opsonized with mouse serum for five min at 37°C. Cells were cultured with FITC-labeled *E. coli* particles (Molecular Probes) for the indicated times, and fluorescence was analyzed with the FACSCanto. To assess bacterial survival, cells were cultured (5 x 10^4^ cells/well) in 96-well plates and infected with five-fold serially diluted *E. coli* (DH5, 4.8x10^5^/well) or *S. aureus* (FDA209, ATCC 6538) in 100 µL RPMI medium containing 10% FBS and 3% GM-CSF-CM at 37°C for 16 hr. After infection, the culture medium of optimally diluted wells was plated on LB (for *E. coli*) or nutrient broth (for *S. aureus*) agar plates and incubated at 37°C overnight. The number of colonies was evaluated.

### Microarray

RNA was extracted from GM-IMs or BMDCs with the RNeasy mini kit. Biotin-labeled cRNA was synthesized from 250 ng total RNA and ten µg cRNA were used for hybridization with the GeneChip mouse Genome 430 2.0 array by using the GeneChip3’IVT Express Kit and Hybridization, Wash and Stain kit according to the manufacturer’s manual (Affymetrix). Raw data were analyzed with Affymetrix GeneChip Command Console Software and Affymetrix Expression Console Software by Takara Bio.

### Macrophage reconstitution

Macrophage depletion was accomplished with injection of clodronate as described [18]. Clodronate liposomes (Clodronate Liposomes Foundation (Netherlands)) were injected intravenously into C75BL/6 mice (50 mg in 200 µL PBS per mouse) to deplete endogenous tissue macrophages. Two days later, GM-IMs (1 x 10^6^) were injected intravenously into the mice.

### Histological analysis

The spleens from mice were embedded in O.C.T compound (Sakura Finetek). The sections were fixed with 4% paraformaldehyde and stained with hematoxylin and eosin. For immunohistochemistry, the sections were fixed with 4% paraformaldehyde for ten min and were quenched in 1% hydrogen peroxidase/methanol for 20 min. Blocking was done in PBS containing 1.5% nonfat dry milk and 0.3% Triton-X for one hr, followed by incubation with the primary antibodies, rat anti-F4/80 (BMA, 1:100) in blocking buffer overnight at 4°C. After incubation with HRP conjugated anti-rat IgG (KPL) for two hr, the peroxidase activity was visualized with 3, 3’-diaminobenzidine tetrahydrochloride (Dojindo). After counterstaining with hematoxylin, slides were subjected to an alcohol dehydration series and mounted with malinol. Slides were examined on an Olympus microscope. Images were recorded using Nuance and analyzed with InForm (PerkinElmer).

### Statistical analysis

Results are expressed as means ± S.E. The statistical analysis was carried out by one-way ANOVA followed by Fischer’s PTSD test. P values less than 0.05 were considered statistically significant.

## Results

### 1: Selection of self-renewable GM-CSF-dependent immature macrophages by long-term culture of BM cells with GM-CSF

**Figure 1:**
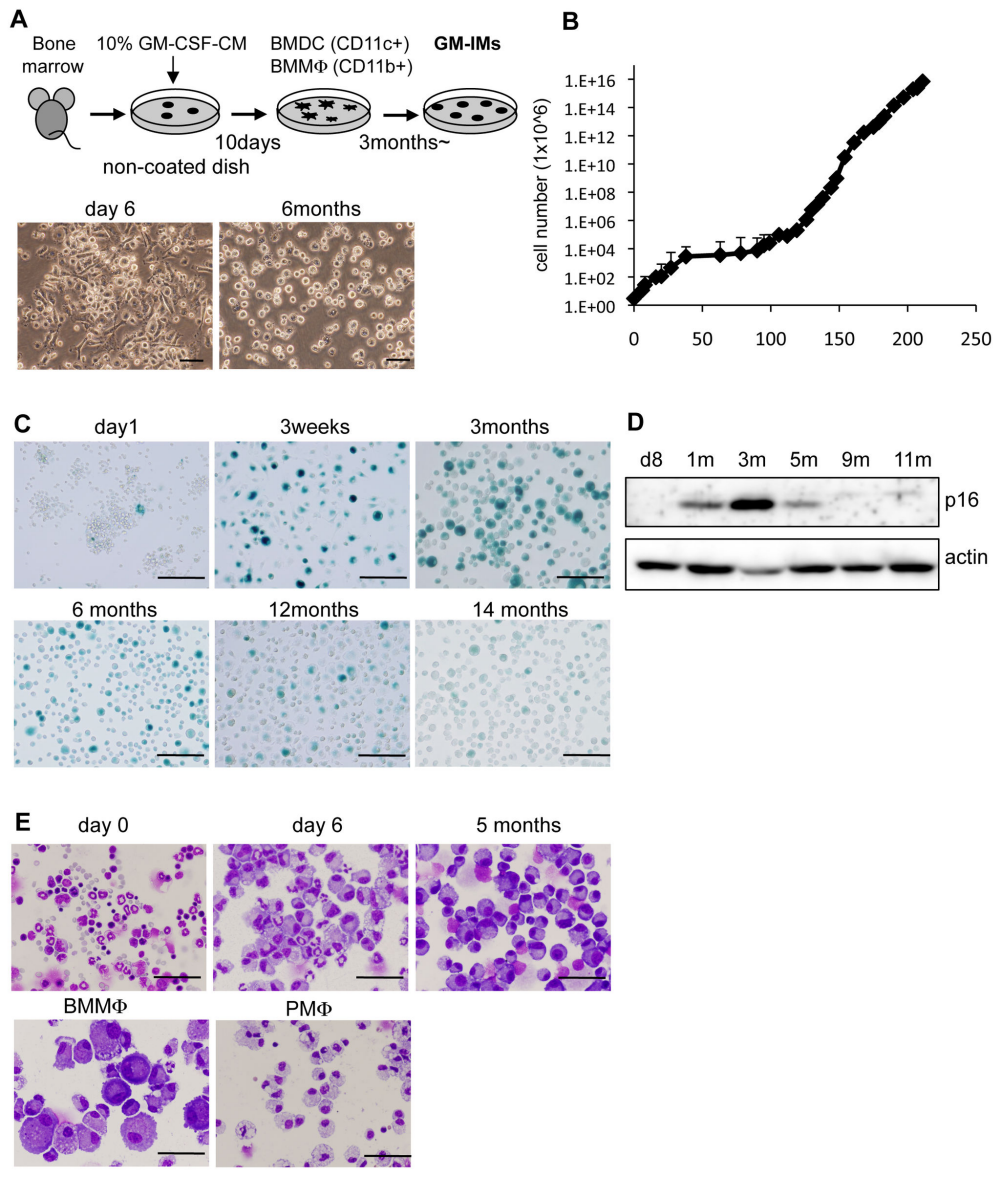
Establishment of GM-IMs from murine BM. (A) BM cells were taken from two-month old C57BL/6 or EGFP mice. Suspensions of BM cells (3x10^6^ cells) were plated in ten cm diameter non-coated plastic culture dishes. They were cultured in RPMI-1640 supplemented with 10% GM-CSF-CM and 10% FBS. Lower panel shows phase contrast photograph of BM cultured with 10% GM-CSF-CM for six days or six months. Scale bars: 50 µm. (B) Growth curve of GM-IMs. BM cells cultured with 10% GM-CSF-CM for the indicated times, during which cells became GM-IMs. Data shown are the mean ratios ± SE of four independent experiments. (C) SA-β-gal staining of BM cells cultured with 10% GM-CSF-CM for the indicated times, during which cells became GM-IMs. Scale bars: 100 µm. (D) Expression of cellular senescent marker proteins. Cell lysates were Western blotted for p16. Β-actin was used as a control. (E) May-Grunwald-Giemsa staining of GM-IMs. Five months cultured GM-IMs was compared with days zero and six of BM culture in 10% GM-CSF-CM, BMMφ and peritoneal macrophages (PMφ). Scale bars: 50 µm. Data are representative of three independent experiments with similar results (A, C-E).

We cultured BM cells from C57BL/6 or EGFP-C57BL/6 mice in the presence of conditioned medium made by cells secreting recombinant murine GM-CSF (GM-CSF-CM). This method is frequently used to prepare murine BMDCs or BMMφ [19]. Early in the cultivation of BM cells in non-coated culture dishes with GM-CSF-CM, we observed mixed populations of myeloid cells (Figure 1A). BMDCs are non-adherent cells, whereas BMMφ are adherent. Both were collected after six to 12 days of GM-CSF culture for these experiments. When these cells were continuously cultured in 10% GM-CSF-CM over the long-term, cells had a round shape and were loosely attached to the non-coated dish (Figure 1A). The growth of cells declined between three weeks to three months and then increased (Figure 1B). Most cells cannot proliferate in GM-CSF and die by cellular senescence. Indeed, the number of cells staining positively for senescence-associated-β galactosidase (SA-β gal) increased from three weeks to three months (Figure 1C). During continuous culture, the staining of SA-β weakened, which correlated with the cell growth curve. The expression of p16^INK4A^ was gradually upregulated and peaked at three months of culture (Figure 1D). It correlated with the stagnation period of growth. During continuous culture, we observed the appearance of a homogenous population of round cells that grew well and were relatively small compared with BMMφ (Figure 1E). This cell population could be cultured for more than one year. These long-term cultured myeloid cells were termed GM-CSF-dependent immature macrophages (GM-IMs).

**Figure 2:**
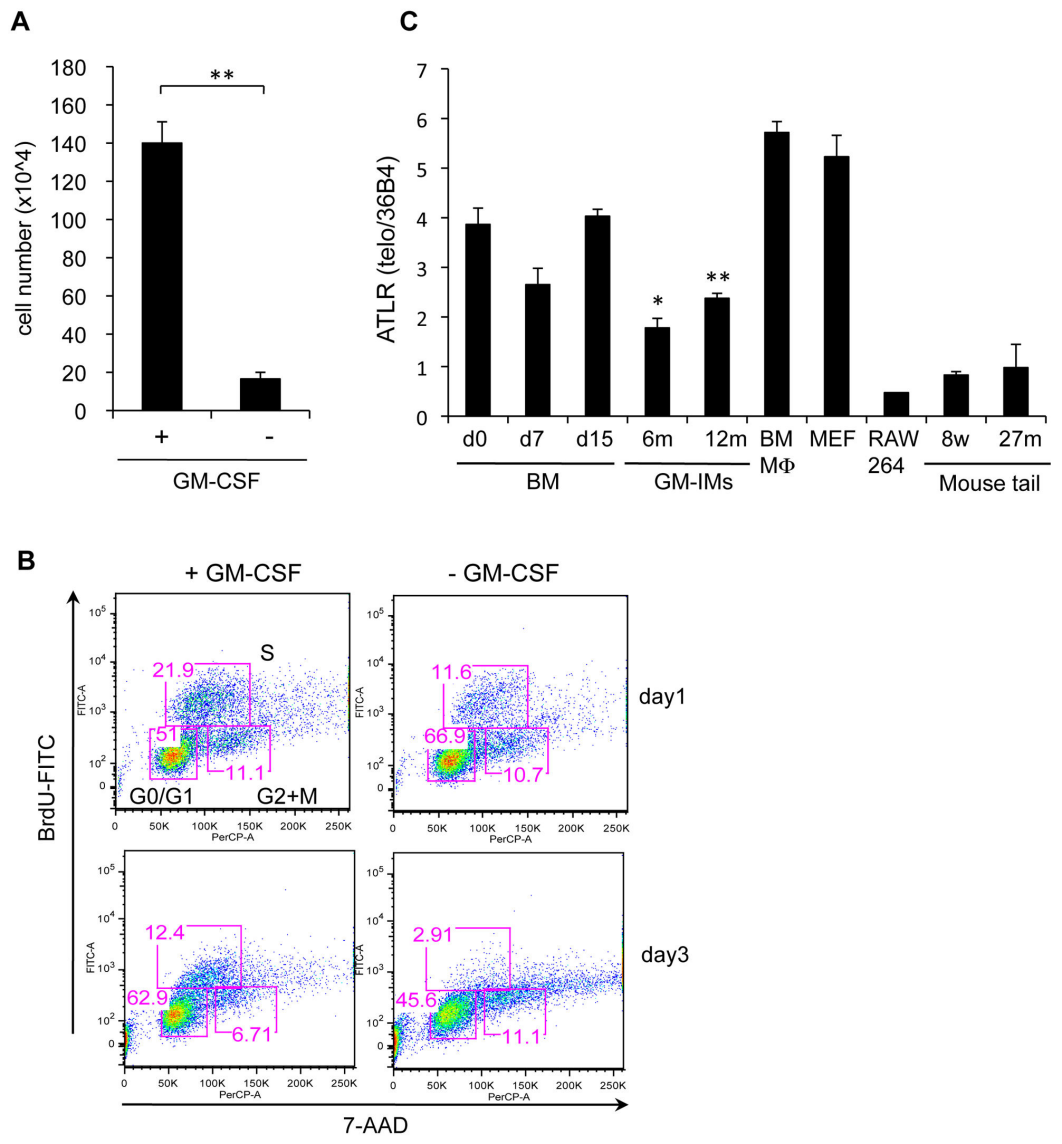
Continuous proliferation of GM-IMs was dependent upon GM-CSF. GM-IMs were plated in ten cm non-coated plastic dishes. They were cultured in RPMI-1640 supplemented with 10% FBS with or without 10% GM-CSF-CM. (A) GM-IMs (3x10^5^) were cultured in ten cm non-coated dishes and after six days of culture, the cell number was counted by trypan blue dye exclusion. Data shown are the mean ratios ± SE of four independent experiments. P value: **<0.001. (B) On days one and three of culture, cells were stained with BrdU-FITC and 7-AAD and analyzed by flow cytometry. Data are representative of three independent experiments. (C) Comparison of the average telomere length ratios (ATLR) between BM cells cultured in 10% GM-CSF-CM for the indicated times during which cells became GM-IMs and indicated cells or murine tails. Data shown are the mean ratios ± SE of three independent experiments. P value: *<0.01, **<0.001 in comparison to that of tail from 8 weeks old murine.

It is possible that during cellular senescence, oncogenes were activated and the GM-IMs became tumor-like cells. To exclude this possibility, GM-IMs were cultured with or without GM-CSF-CM. GM-IMs cultured without GM-CSF-CM did not increase and died (Figure 2A) and no active cell cycling was observed in the absence of GM-CSF-CM (Figure 2B). In the presence of GM-CSF-CM, GM-IMs have low amount of p53 and p21expression during long-term culture. However methyl methanesulfonate (MMS) treatment induced the expression of p53 and p21 in GM-IMs (Figure S1A). These results indicated that the proliferation of GM-IMs depended on GM-CSF and that GM-IMs were not transformed cells.

Although human somatic cells undergo telomere shortening, long-term culture of somatic murine cells is not limited by telomere length and we observed the maintenance of telomere length. Although the length of GM-IMs telomeres was less than those of MEF or BMMφ, it was much greater than those of mouse-tails and RAW264.7 cells (Figure 2C). Also, *Tert* expression, which maintains telomere length, did not decline during long-term culture (Figure S1B).

### 2: Characteristics of GM-IMs

**Figure 3:**
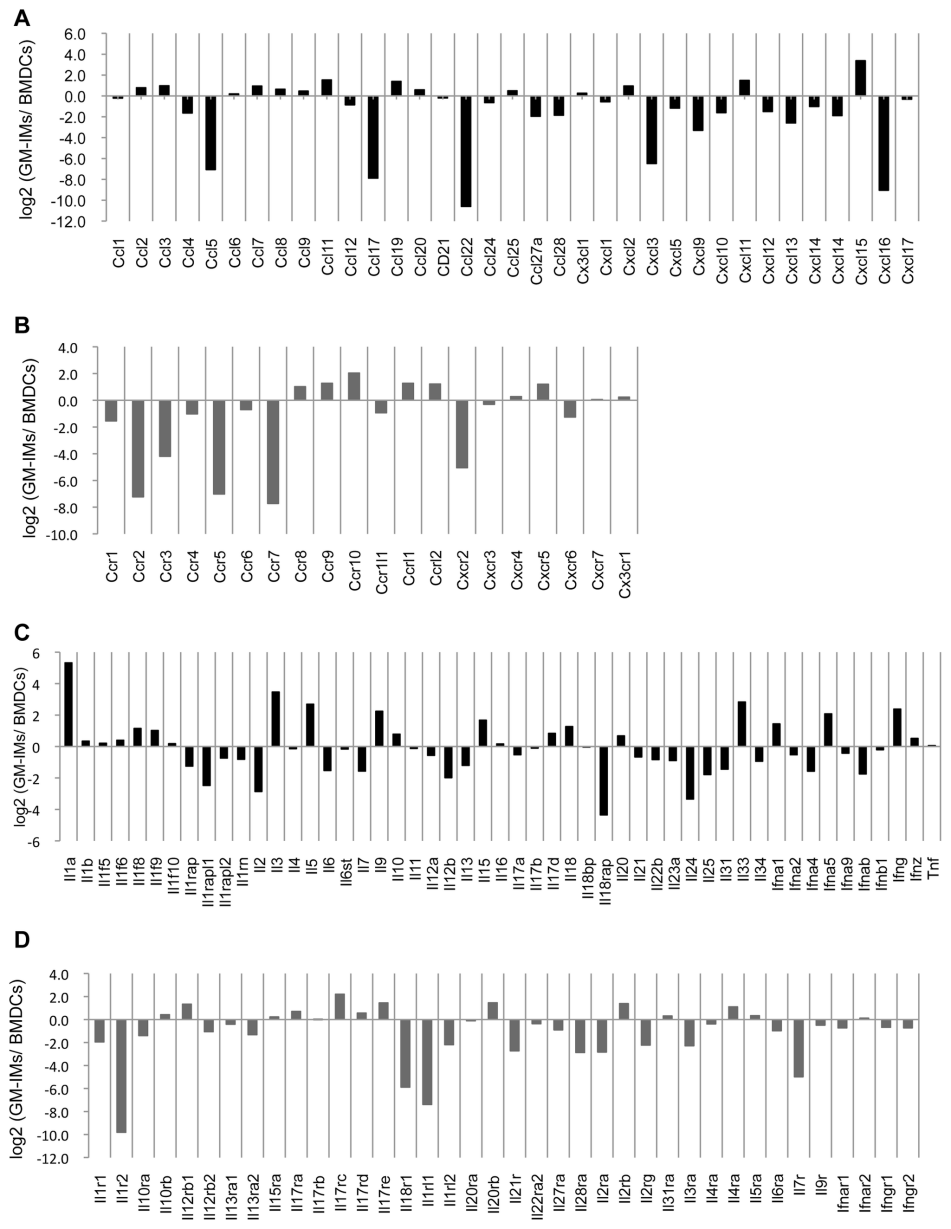
Gene expression profiles of GM-IMs compared to BMDCs. Comparison of gene expression levels between ten months cultured GM-IMs and BMDCs cultured for six days, both in 10% GM-CSF-CM. They were analyzed with a GeneChip mouse genome 430 2.0 microarray. (A) Relative chemokine expression of GM-IMs compared to BMDCs. (B-D) Relative chemokine receptor (B), cytokine (C) and cytokine receptor (D) expression, as in (A).

We examined the gene expression profiles of GM-IMs and BMDCs because both grew in GM-CSF-CM. The expression of several chemokine genes (*Ccl5*, *Ccl17*, *Ccl22*, *Cxcl3* and *Cxcl16*) was lower in GM-IMs than in BMDCs (Figure 3A). These chemokines are produced by activated macrophage lineage cells and attract macrophages and lymphocytes. Many chemokine receptors on macrophage lineage cells (*Ccr2*, *Ccr3*, *Ccr5*, *Ccr7* and *Cxcr2*) were lower in GM-IMs than in BMDCs (Figure 3B). GM-IMs expressed *Il1a*, *Il3*, *Il5*, *Il9*, *Il15* and *Il33* genes at higher levels than those in BMDCs, although many other cytokine genes were expressed at lower levels (Figure 3C). Also, expression of cytokine receptors on GM-IMs was lower than those on BMDCs (Figure 3D). Thus, although the same groups of genes were expressed in GM-IMs and BMDCs, many were expressed at different levels (Figure S2).

**Figure 4:**
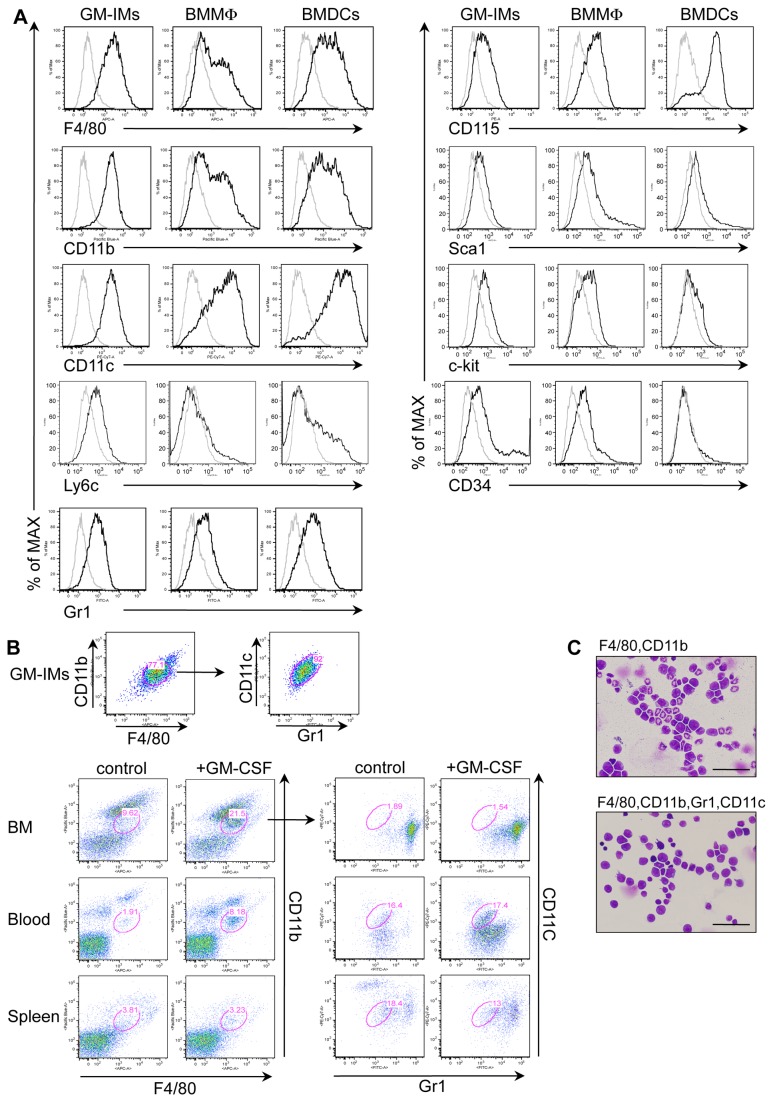
Expression of macrophage lineage makers on GM-IMs. (A) GM-IMs cultured for more than nine months in 10% GM-CSF-CM were compared with BMMφ or BMDCs cultured for ten days in 10% GM-CSF-CM. They were collected and stained with indicated antibodies and analyzed by flow cytometry using a FACSCanto. (B) BM cells, blood nucleated cells and splenocytes were collected from control mice and mice that had been injected intraperitoneally twice (once per week) with three mL of 10% GM-CSF-CM and were analyzed after two weeks. These cells and GM-IMs were detected by F4/80 and CD11b expression and were gated to assess CD11c and Gr-1. (C) C57BL/6 mice were injected once with three mL of 10% GM-CSF-CM. After seven days, BM cells were prepared and stained with FITC-anti-Gr1, APC-anti-F4/80, eFluor450-anti-CD11b and PE-Cy7-anti-CD11c. Stained cells were sorted with a wide gate (CD11b and F4/80) or a narrow gate (F4/80, CD11b, CD11c, Gr-1). Cells underwent Cytospin preparation and were stained with May-Grunwald-Giemsa. Scale bars: 50 µm. Data are representative of three (A, B) or two (C) independent experiments with similar results.

We examined cell surface markers of GM-IMs. GM-IMs stained positively for an F4/80^high^ CD11b^high^ CD11c^low^ Ly6C^low^ Gr-1^low^ phenotype (Figure 4A). Because GM-IMs are a homogenous population after long-term cultivation in GM-CSF-CM, we examined whether the same population existed in the hematopoietic system. We injected GM-CSF-CM twice into mice to determine if the same population of GM-IMs could be expanded. We found the same level of expression of F4/80**^+^** CD11b^+^ double positive cells in BM, blood and spleen of control and GM-CSF-CM-injected mice as that of GM-IMs. GM-CSF-CM injection increased the percentage of cells that had the same level of F4/80 and CD11b expression as GM-IMs. Because these included other cells than GM-IMs, which expand in the presence of GM-CSF-CM, we narrowed the gate using two additional markers, CD11c and Gr-1. We found that a population similar to GM-IMs was present, both unstimulated and stimulated in BM, blood and spleen (Figure 4B). Furthermore, we sorted BM using a wide gate (F4/80 and CD11b) or a narrow gate (F4/80, CD11b, Gr-1, CD11c). The cell population in wide gate contained not only GM-IMs-like monocyte cells but also other myeloid cells such as BMDCs, BMMφ and neutrophil. In contrast, the cell population found in the narrow gate contained only GM-IMs-like cells (Figure 4C). Furthermore, the sorted cells obtained in the narrow gate proliferated well in 10% GM-CSF-CM and gradually became a single population with characteristics of GM-IMs (data not shown). These results suggest that GM-IMs are present in BM.

**Figure 5:**
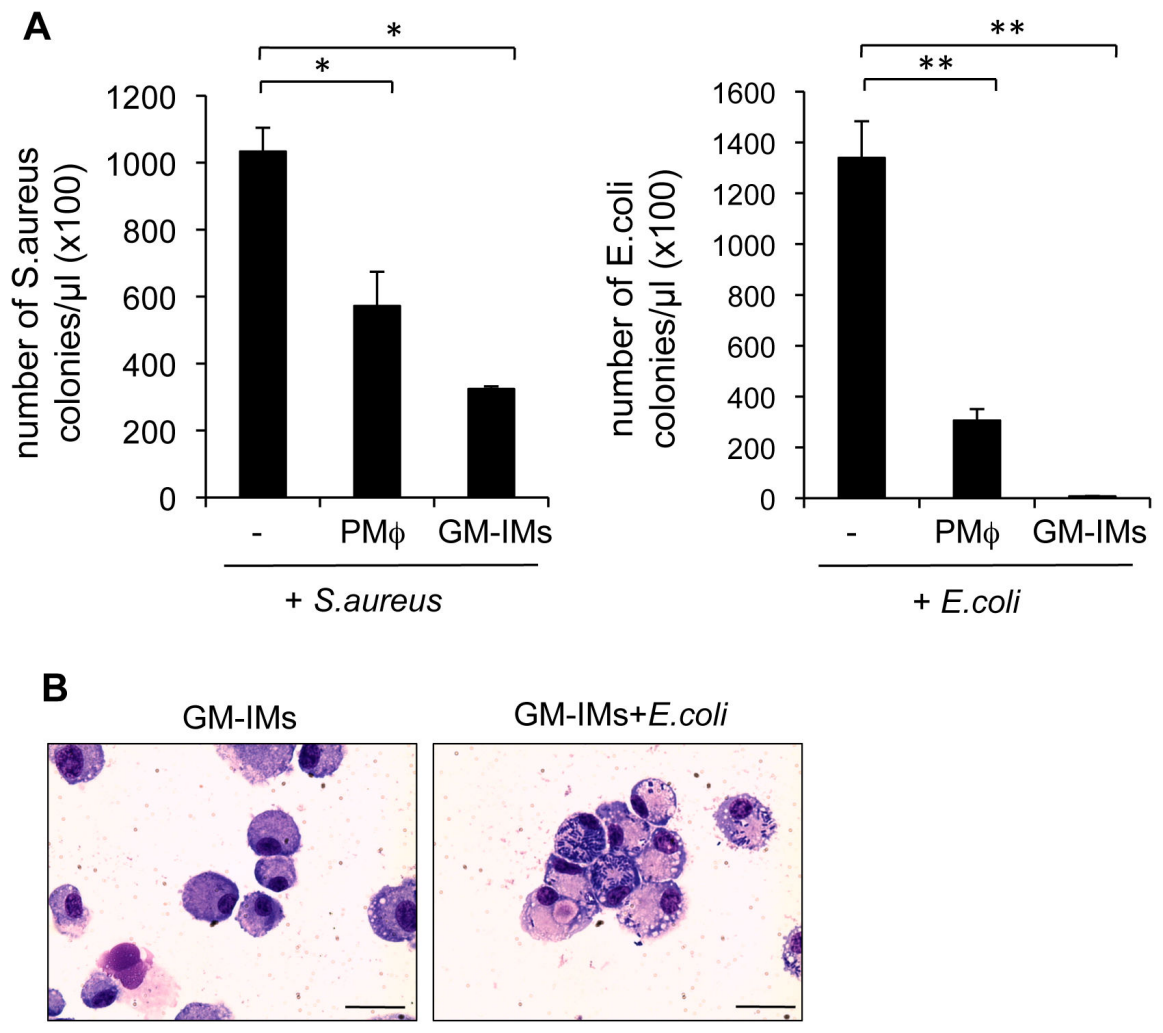
Functional analysis of GM-IMs. (A) Bacterial killing assay. *S. aureus* (left) or *E. coli* (right) were serially diluted in 96-well plates. They were mixed with 5 x 10^4^ PMφ or GM-IMs and cultured overnight in 100 µL RPMI-1640 and 10% FCS and 3% GM-CSF-CM. One µL of mixed culture at appropriate dilution (which showed the clear differences in the number of bacteria) was plated on an agar plate. After 24 hr, the number of colony forming units (CFU) was counted. Data shown are the mean ratios ± SE of three independent experiments. P value: * <0.01, **<0.001. (B) May-Grunwald-Giemsa stain of GM-IMs cultured with *E. coli* for 24 hr. Left shows GM-IMs without bacteria, and right shows GM-IMs with bacteria. Scale bars: 100 µm. Data are representative of three independent experiments.

Next, we analyzed the function of GM-IMs. The phagocytic ability of GM-IMs was compared with peripheral macrophages. GM-IMs had a higher ability to kill *Staphylococcus aureus* (*S. aureus*) and *Escherichia. coli* (*E. coli*) than did peripheral macrophages (Figure 5A). Phagocytosis of FITC-labeled *E. coli* by GM-IMs was analyzed by flow cytometry. The phagocytic activity of GM-IMs was increased by opsonization (Figure S3). By Giemsa staining, GM-IMs contained a high number of *E. coli* after one day of co-culture (Figure 5B).

**Figure 6:**
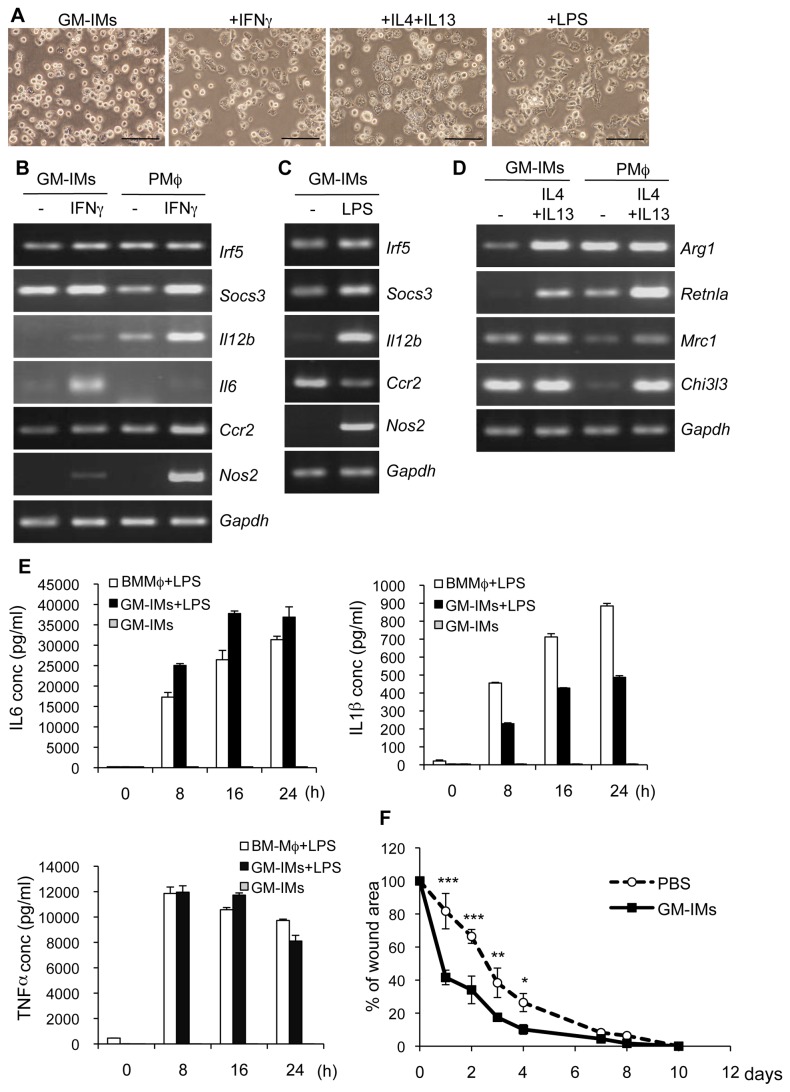
Potentials of GM-IMs to differentiate to M1 or M2 macrophages. GM-IMs or PMφ were stimulated with 20 ng/mL IFNγ, 20 ng/mL IL-4 and 20 ng/mL IL-13 or ong µg/mL LPS for 24 hr. (A) Phase contrast photographs of GM-IMs after stimulation. Scale Bar: 100 µm. (B) After stimulation by IFNγ for 24hr, expression of M1 marker genes was analyzed by RT-PCR. (C) After stimulation by LPS, RNA was extracted and expression of M1 marker genes was analyzed by RT-PCR. (D) After stimulation by IL-4 and IL-13, expression of M2 marker genes was analyzed by RT-PCR. (E) GM-IMs or BMMφ (1 x 10^6^) were cultured in six-well plastic plates with RPMI-1640 containing 10% FBS and 3% GM-CSF-CM. They were stimulated with one µg/mL LPS for indicated times. Supernatants were taken and cytokine expression of IL-6, IL-1β or TNFα was analyzed by ELISA. Data shown are the mean ratios ± SE of three separate experiments. (F) The dorsal skin of two-month old female C57BL/6 mice (n=4) was punctured through two layers of skin with a sterile disposable three mm biopsy punch (Kai Industries). GM-IMs (3 x 10^4^) were directly injected into each wound on the right side. PBS was dropped on the other side as a control. The changes in the percentages of wound areas at each time point were compared to the initial wound area. Data shown are the mean ratios ± SE of three independent experiments. P value: * < 0.05, **<0.01, ***<0.001, GM-IMs injected area versus PBS injected area. Data are representative of three independent experiments with similar results (A-D).

We examined whether GM-IMs could differentiate into M1 or M2 macrophages. We stimulated GM-IMs with IFNγ (M1 conversion) or IL-4 + IL-13 (M2 conversion). After stimulation by IFNγ or IL-4 and IL13, the round monocyte-like GM-IMs converted to flat shaped macrophage-like cells and attached to the plastic dish (Figure 6A). IFNγ enhanced GM-IMs’ expression of M1 marker genes (*Irf5*, *Socs3*, *Il12b, Il6, Ccr2* and *Nos2*), although the increase of the expression of these genes was lower than that of peritoneal macrophages (Figure 6B). GM-IMs expressed IL-6 following stimulation by IFNγ. In order to examine whether GM-IMs were activated to produce cytokines, we stimulated GM-IMs with LPS, a Toll-like receptor ligand that converts monocytes to M1 macrophages. GM-IMs differentiated to flattened macrophage-like cells and attached to the plastic dish after LPS treatment (Figure 6A). LPS also enhanced M1 marker genes in GM-IMs (Figure 6C). This treatment induced production of TNFα, IL-6 and IL-1β in GMIMs as well as in BMMφ (Figure 6E). These results indicated that GM-IMs converted to M1 macrophages *in vitro* following stimulation by IFNγ or LPS. After stimulation by IL-4 and IL-13, expression of *Arg1* and *Retnla* was highly enhanced in GM-IMs. GM-IMs expressed *Mrc1* and *Chi3l3* without and with stimulation by IL-4 and IL-13 (Figure 6D). These results show that GM-IMs converted to M2 macrophages after treatment with IL-4 and IL-13. We examined whether GM-IMs could affect wound healing. Injection of GM-IMs into mouse dorsal skin accelerated repair (Figure 6F). The expression of *Chi3l3* was statistically enhanced in the wound site where GM-IMs were injected compare with PBS injected (Figure S4). These characteristics of GM-IMs are similar to those of M2 macrophages. Taken together, GM-IMs can be converted to M1 or M2 macrophages depending on the stimuli chosen for treatment.

GM-IMs did not produce IL-12 after stimulation with LPS (Figure S5A). Although BMDC and BMMφ increased their expression of MHC class II and the T cell costimulator molecule CD86 after stimulation by LPS, GM-IMs showed only slight increases of these molecules following LPS stimulation (Figure S5B). These results indicated that GM-IMs could not differentiate to antigen presenting cells after LPS stimulation. GM-IMs did not secrete IL-10 after stimulation with LPS, a characteristic of regulatory macrophages (Figure S5A).

### 3: GM-IMs enter macrophage-depleted sites and induce proliferation of resident macrophages

**Figure 7:**
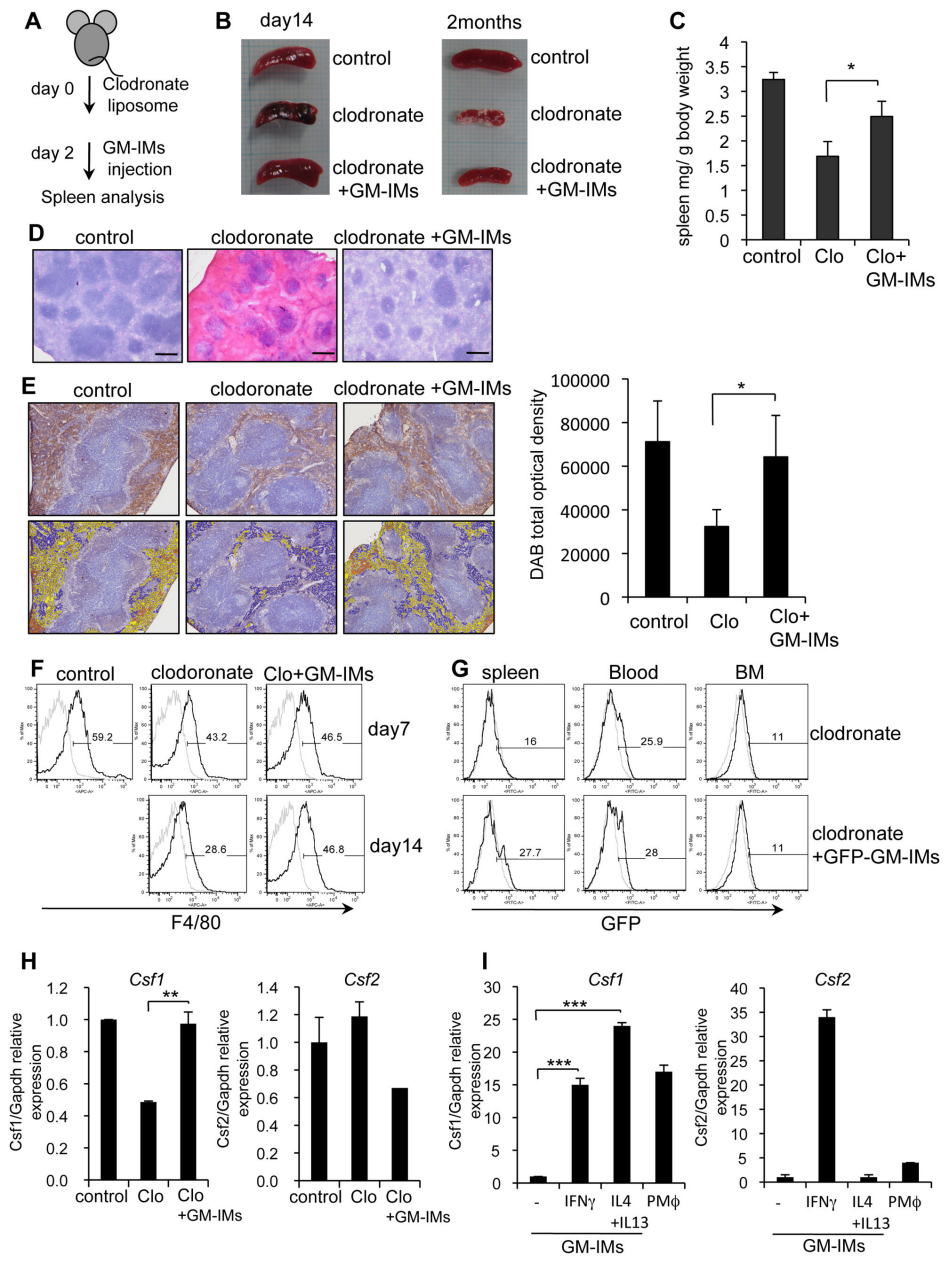
Effects of injection of GM-IMs to clodronate-liposome treated mice. (A) Schematic outline of clodronate-liposome and GM-IMs treatment. Two-month old female C57BL/6 mice were intravenously injected with clodronate liposomes. After two days, 5 x 10^6^ GM-IMs were injected intravenously. (B) Spleens were taken after 14 days or two months of clodronate treatment. (C) The spleen weight per body weight of mice for day 15 to one month after clodronate-treatment. Data shown are the mean ratios ± SE of seven mice. (D) Frozen sections of spleens two months after clodronate treatment were stained with H and E. Scale Bars: 300 µm. (E) Frozen sections of spleen two months after injection of clodronate were assessed for F4/80 expression by DAB staining (upper). Colors indicate the strength of DAB staining: orange is highest, yellow is mid-level and blue is lowest (lower). Total DAB optical densities are the mean rations ±SE of three independent experiments. (F) The expression of F4/80 in the spleen on days seven or 14 after injection of clodronate. F4/80 was detected by flow cytometry. (G) GM-IMs from EGFP-C57BL/6 mice were injected intravenously after two days of clodronate injection. GFP-positive tissue cells were analyzed by flow cytometry. (H) Expression of *Csf1* and *Csf2* in the spleen. Spleens were taken from C57BL/6 mice after seven days of clodronate injection or clodronate and GM-IMs injection. The expression of *Csf1* and *Csf2* was analyzed by real-time RT-PCR. The data were normalized to *Gapdh*. (I) Expression of *Csf1* and *Csf2* in stimulated GM-IMs and PMφ. GM-IMs were stimulated with 20 ng/mL IFNγ or 20 ng/mL IL-4 and 20 ng/mL IL-13 for 24 hr. The expression was analyzed as in (H). Data shown are the mean ratios ± SE of three independent experiments (H, I). P value: *<0.05, **<0.01, ***<0.001.

Because GM-IMs are derived from BM cells, a population that contains HSCs, we hypothesized that GM-IMs might represent a stage of myeloid lineage development and that GM-IMs might support the recovery of tissue macrophages after their depletion. Clodronate is known to deplete tissue macrophages. We injected GM-IMs into clodronate liposome-treated mice (Figure 7A). When mice were treated with clodronate liposomes, we observed that spleen size decreased. Following intravenous injection of GM-IMs into clodronate-treated mice, spleen size recovered (Figure 7B, C). Whereas clodronate-treatment decreased the number of cells in the red pulp, injection of GM-IMs reversed the effect (Figure 7D). Clodronate-treatment decreased F4/80^+^ macrophages in the spleen, but recovery was seen after two months of GM-IMs injections (Figure 7E), with an increase after two weeks (Figure 7F, S6A). F4/80^+^ cells slightly increased in normal spleens after GM-IMs injection (Figure S6B). We initially thought that GM-IMs might have proliferated in the macrophage-depleted spleens. However, when we injected GM-IMs taken from EGFP-C57BL/6 mice, we noted only a brief increase of GFP-positive cells in the spleen and almost all macrophages were of host origin (Figure 7G, S6C). These data indicated that GM-IMs might have entered the clodronate-depleted spleens and induced the proliferation of resident host macrophages but not GM-IMs. Because macrophage proliferation is supported by M-CSF, we measured *Csf1* (encoding M-CSF) expression in spleen. Clodronate-treated spleens decreased *Csf1* expression, which reduced the resident macrophages. After the injection of GM-IMs, we found high expression of *Csf1* in the spleen (Figure 7H). However the expression of *Csf2* (encoding GM-CSF) was not changed in spleen after treatment of clodronate and the injection of GM-IMs. Also GM-IMs increased the expression of *Csf1* after stimulation by IFNγ or IL-4 and IL-13 *in vitro* (Figure 7I). We noted that GM-IMs had a low level of M-CSF receptor expression and they did not increase in the presence of M-CSF-CM (Figure 4A, data not shown). These results suggest that GM-IMs are unable to proliferate in spleen and resident macrophages inside the spleen may grow with M-CSF produced by GM-IMs.

## Discussion

There have been no reports of the establishment of normal hematopoietic cells capable of proliferating long-term in vitro in the absence of a feeder layer. Here, we demonstrated that long-term cultivation of myeloid lineage cells could be achieved. Because GM-IMs can self-renew in the presence of GM-CSF, we propose that the same myeloid population might exist *in vivo*. We suggest that GM-CSF continuously produced *in vivo* could support a small population of myeloid lineage cells that self-renews. When the level of GM-CSF increases, this population would be expected to increase.

The GM-IMs described here are part of the MPS because GM-IMs phagocytize and kill *E. coli* and *S. aureus*. Monocytes, DCs, macrophages, neutrophils and mast cells are all professional phagocytes and they originate from BM HSCs. At present, it is not clear at what stage of myeloid differentiation GM-IMs appear. Several models have been proposed in which HSCs differentiate to either myeloid or lymphoid lineages. However, the developmental branching points between lymphoid and myeloid lineages are still controversial [20]. Weissman’s group found that all myeloid (including erythroid and megakaryocytic) cells arise from common myeloid progenitors (CMPs) [21]. CMPs subsequently develop into more restricted progenitors, such as granulocyte/monocyte progenitors (GMPs) [21]. Katsura et al. proposed a myeloid-based model of hematopoiesis in which myeloid potential is retained at an early stage of development and branches toward erythroid, T-, and B-cell lineages [22]. GM-IMs likely arise after the appearance of CMPs proposed by Akashi et al. [21] and after the development of a common macrophage and erythroid progenitor as proposed by Katsura [23]. GM-IMs cells are round and loosely attach to non-coated dishes. GM-IMs have a capacity to phagocytize and kill bacteria. GM-IMs can differentiate to M1 and M2 macrophages. These results indicate GM-IMs belong to a monocyte lineage. In circulating blood, there are two types of monocytes: inflammatory monocytes and patrolling monocytes. LY6C^hi^ inflammatory monocytes express CC-chemokine receptor 2 (CCR2) and can be rapidly mobilized to inflammatory sites [2]. LY6C^low^ patrolling monocytes express high levels of CX3C-chemokine receptor 1 (CX3CR1) and lack the expression of CCR2. The spleen harbors large numbers of LY6C^hi^ monocytes in the subcapsular red pulp from which they rapidly emigrate to inflammatory sites [24]. Expression of CCR2 and CX3CR1 is low in GM-IMs. Thus, GM-IMs are neither inflammatory nor patrolling monocytes. GM-IMs express low amounts of CCR3, a CCR that is selectively expressed on eosinophils, basophils, and some Th2 cells [25]. GM-IMs also lack the expression CXCR2, which is a receptor for CXCL1, -2, -3, -5, -7 and -8 [26]. These results indicate that GM-IMs are relatively quiescent [27].

Monocytes differentiate to macrophages that reside in all tissues and protect the body from pathological insults. They are also involved in the pathogenesis of various diseases [3]. Macrophages are subdivided into M1 and M2 classes [14,28]. GM-IMs differentiate to M1 macrophages following stimulation by LPS or IFNγ. GM-IMs can also differentiate to M2 macrophages after stimulation by IL-4 and IL-13. GM-IMs can also promote the repair of tissue after injection into a wound site of syngeneic mice. IL-4 and IL-13 are induced by injury or helminth infections. Those cytokines stimulate arginase activity in macrophages, allowing them to convert arginine to ornithine, a precursor of polyamines and collagen, thereby contributing to the production of the extracellular matrix [29]. When GM-IMs are stimulated by IL-4 and IL-13, *Arg1* expression was enhanced. Wound healing macrophages produce chitinase and chitinase-like molecules YM1(*Chi3l3*) and FIZZ1(*Retnla*) [30]. *Chi3l3* was expressed at high levels by GM-IMs and *Retnla* was upregulated after stimulation by IL-4 and IL-13. These data suggest that GM-IMs can differentiate to M2 macrophage. GM-IMs are not regulatory macrophages because GM-IMs do not secrete IL-10, when stimulated by LPS.

Recent findings show that adult tissue macrophages are primarily derived from the YS in the early embryo. This finding suggested that monocytes derived from bone marrow HSCs constitutively generate tissue-resident macrophages [12,31-33]. However, the relationship between macrophages originating from circulating monocytes and resident macrophages has not been elucidated. Here, we showed here that F4/80 ^+^ macrophage was reduced in spleen by the injection of clodronate. GM-IMs injection led to recovery of F4/80 ^+^ macrophage. However, GM-IMs did not proliferate in spleen. A recent report showed that tissue resident macrophages, including those in the spleen, which come from YS, can self-renew [12]. Furthermore, Hashimoto et al. showed that lung macrophages self-renew in a M-CSF- and GM-CSF-dependent manner [34]. Our data showed that M-CSF and GM-CSF are constantly expressed in the spleen, which may induce self-renewal of resident macrophages. Following injection of clodronate, expression of M-CSF decreases, although the expression of GM-CSF is not changed. GM-IMs injection increased the expression of M-CSF in the spleen, which might indicate that resident macrophages can recover from some insults. We suggest that GM-IMs might help resident macrophages in the spleen to recover, although further experiments are needed. Here we showed successful long-term culture of immature macrophages in GM-CSF (GM-IMs). GM-IMs differentiated to M1 and M2 macrophages after stimulation respectively by IFNγ or LPS, and IL-4 and IL-13. GM-IMs will be useful for the analysis of macrophage functions *in vitro* and in animal models.

## Supporting Information

Figure S1 **(A) The expression of p53 or p21.** BM cells were cultured with 10% GM-CSF-CM for the indicated times, during which cells became GM-IMs. GM-IMs were treated with 80 µg/mL MMS (Sigma) for six hr. Expression of phospho-p53 or p53 protein was detected by Western blotting (upper). Antibodies to phospho-p53 (Ser15), p53 and GAPDH were from Cell signaling. The expression of the p21 was measured by real-time PCR (lower). The data were normalized to *Gapdh*. Data are representative of three independent experiments.
(B) *Tert* expression. RNA was extracted from BM cells cultured in 10% GM-CSF-CM for the indicated times, during which cells became GM-IMs, BMMφ or MEF. RT-PCR was conducted to assess the expression of *Tert*. Β-actin was used as the internal control.
(TIF)

Figure S2 **Microarray analysis of GM-IMs compared with BMDCs.** The heat map represents log_2_ intensity.
(TIF)

Figure S3 **Flow cytometric analysis of FITC-*E. coli* ingested by GM-IMs.** (A) GM-IMs were cultured with opsonized FITC-E*. coli* particles for 30 min (gray solid peak) and one hr (black line).
(B) GM-IMs were cultured with non-opsonized (gray solid peak) or opsonized (black line) FITC-E*. coli* particles for one hr.
(TIF)

Figure S4 **M2 marker gene expression at the site of GM-IMs injection.** The dorsal skin of two-month-old female C57BL/6 mice (n = 3) was punctured through two layers of skin with a sterile disposable three mm biopsy punch. GM-IMs (3 x 10^4^) were directly injected into each wound on the right side. After four hr, the skin around the wound area was collected and RNA was extracted. The expression of M2 marker genes was analyzed by real-time RT- PCR. Data shown are the mean ratios ± SE of three separate experiments. P value: *<0.05.
(TIF)

Figure S5 **Markers of antigen presenting cells and regulatory macrophages.** (A) GM-IMs or BMMφ (1 x 10^6^) were cultured in six-well plates with RPMI-1640 containing 10% FBS and 3% GM-CSF-CM. They were stimulated with one µg/mL LPS for indicated times. Supernatants were taken and expression of IL-12 or IL-10 was analyzed by ELISA. Data shown are the mean ratios ± SE of three independent experiments.
(B) GM-IMs, BMDCs or BMM were stimulated with one µg/mL LPS. After 16 hr, they were stained with FITC-labeled anti-H-2d or FITC-labeled anti-CD86. They were analyzed by flow cytometry. Data are representative of three independent experiments.
(TIF)

Figure S6 **Injection of GM-IMs into macrophage-depleted mice.** (A) Two-month-old female C57BL/6 mice were intravenously injected with clodronate liposomes. After two days, 5 x 10^6^ GM-IMs were injected intravenously. After five or 14 days or two months, spleen cells were stained with PE-labeled-anti-CD11b and APC-labeled-anti-F4/80 and analyzed by flow cytometry.
(B) GM-IMs were injected into normal mice. At day 14, spleen cells were analyzed by flow cytometry.
(C) GM-IMs from EGFP-C57BL/6 mice were injected intravenously after two days of clodronate injection. GFP-positive cells were analyzed by flow cytometry. The data was shown by dot-blot graph.
Data are representative of three independent experiments (A-C).
(TIF)

Table S1 **Primer sequence used for RT-PCR.**
(DOCX)
