# Supplementary figures and images for: Establishment of Self-Renewable GM-CSF-Dependent Immature Macrophages In Vitro from Murine Bone Marrow
Source: PLoS One. 2013 Oct 4;8(10):e76943. doi: 10.1371/journal.pone.0076943 (PMC3790761; doi:10.1371/journal.pone.0076943)

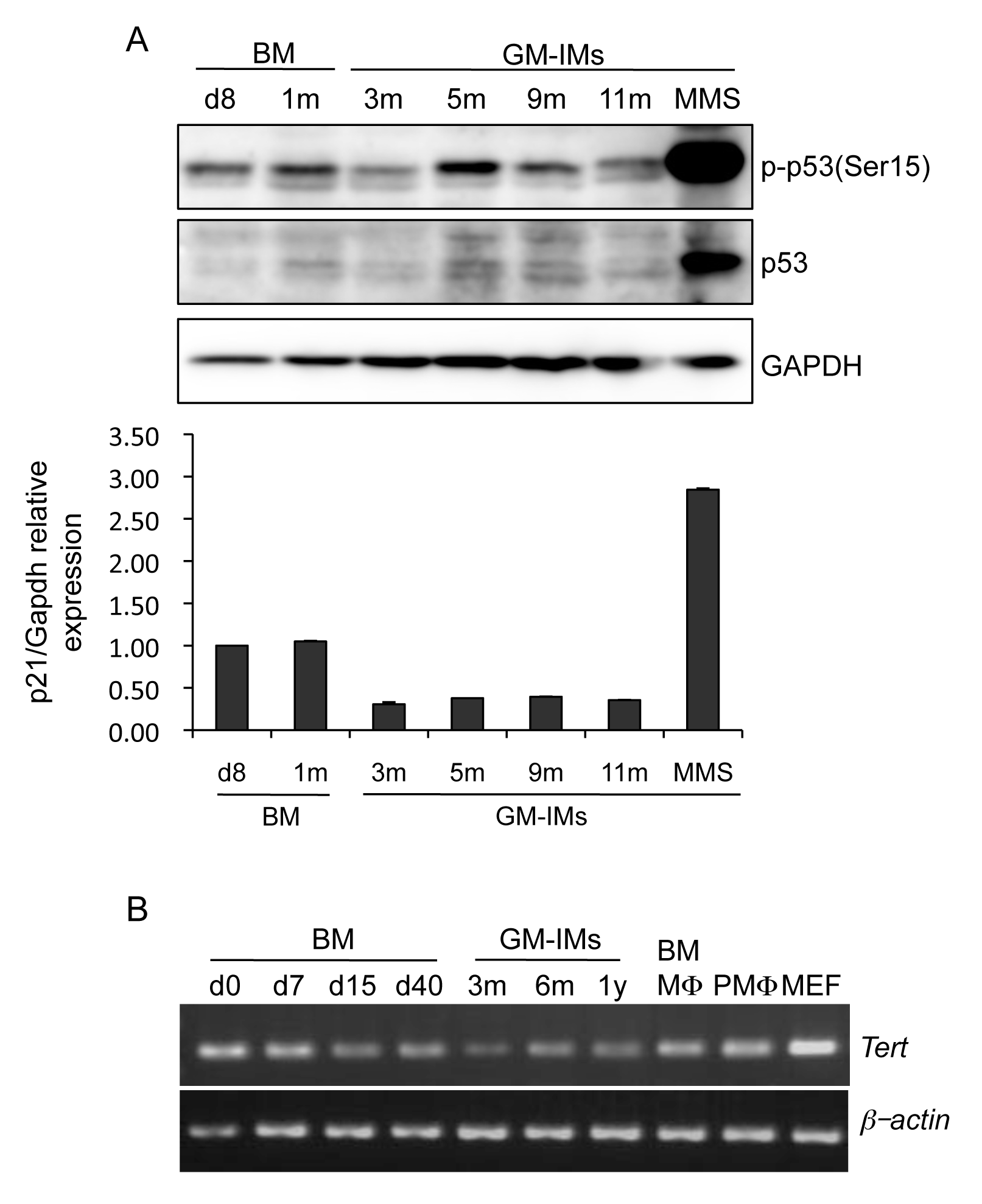

Supplement: Figure S1 — (A) The expression of p53 or p21. BM cells were cultured with 10% GM-CSF-CM for the indicated times, during which cells became GM-IMs. GM-IMs were treated with 80 µg/mL MMS (Sigma) for six hr. Expression of phospho-p53 or p53 protein was detected by Western blotting (upper). Antibodies to phospho-p53 (Ser15), p53 and GAPDH were from Cell signaling. The expression of the p21 was measured by real-time PCR (lower). The data were normalized to Gapdh. Data are representative of three independent experiments. (B) Tert expression. RNA was extracted from BM cells cultured in 10% GM-CSF-CM for the indicated times, during which cells became GM-IMs, BMMφ or MEF. RT-PCR was conducted to assess the expression of Tert. Β-actin was used as the internal control. (TIF) [file pone.0076943.s001.tif]

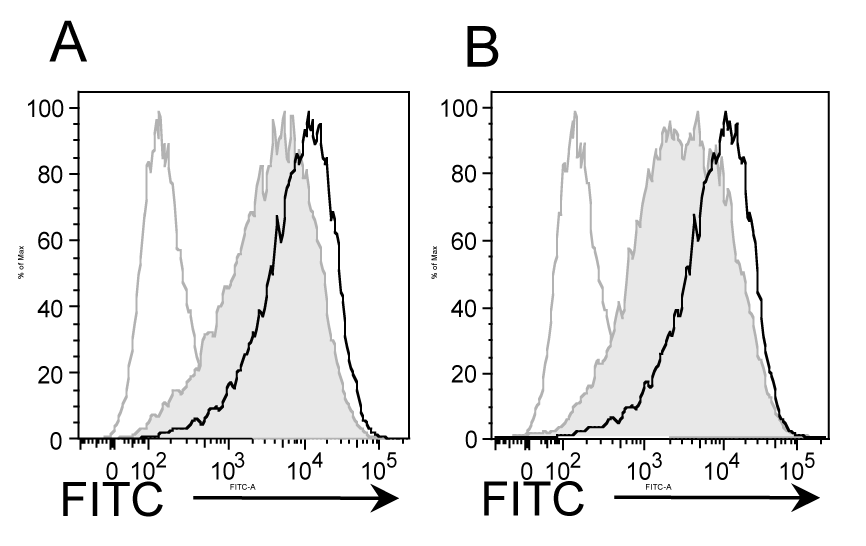

Supplement: Figure S3 — Flow cytometric analysis of FITC-E. coli ingested by GM-IMs. (A) GM-IMs were cultured with opsonized FITC-E. coli particles for 30 min (gray solid peak) and one hr (black line). (B) GM-IMs were cultured with non-opsonized (gray solid peak) or opsonized (black line) FITC-E. coli particles for one hr. (TIF) [file pone.0076943.s003.tif]

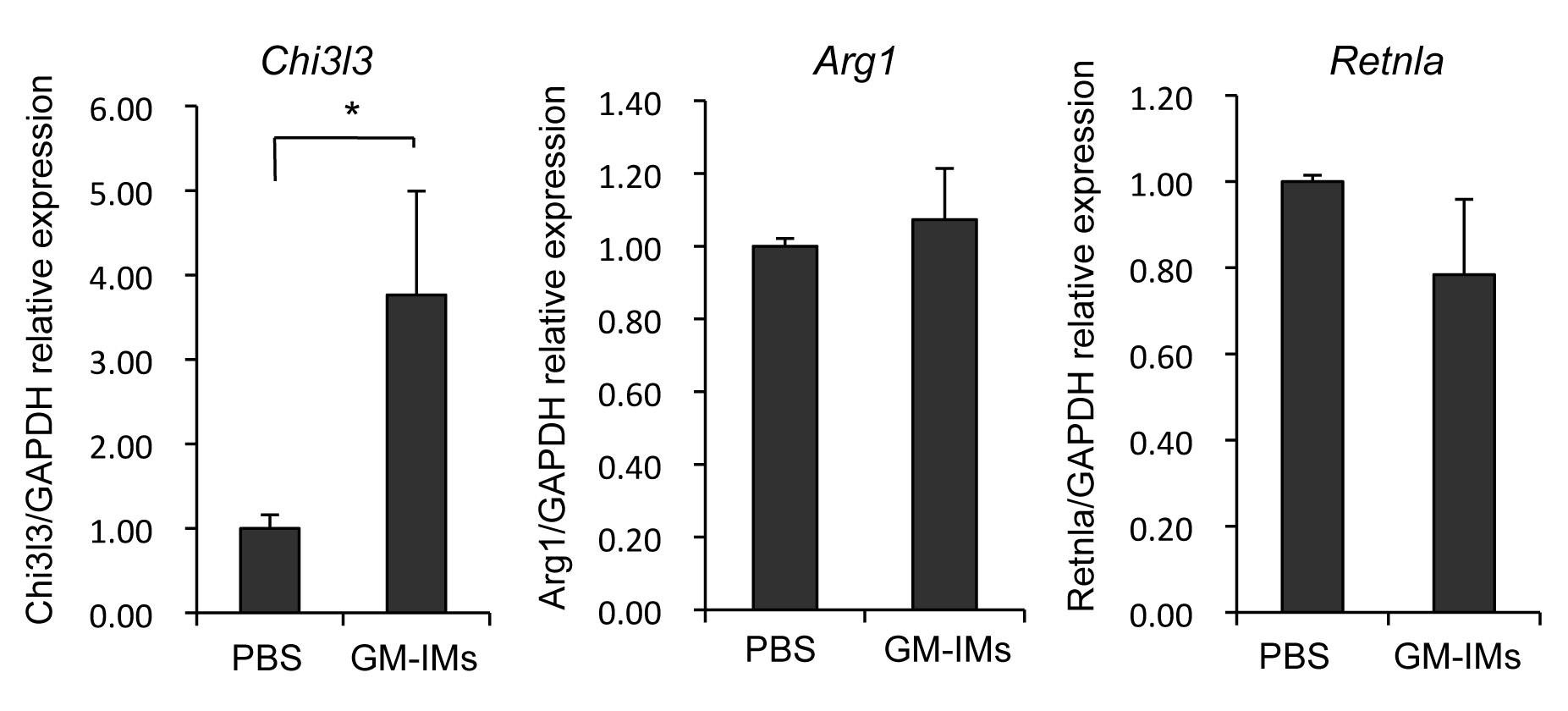

Supplement: Figure S4 — M2 marker gene expression at the site of GM-IMs injection. The dorsal skin of two-month-old female C57BL/6 mice (n = 3) was punctured through two layers of skin with a sterile disposable three mm biopsy punch. GM-IMs (3 x 10^4^) were directly injected into each wound on the right side. After four hr, the skin around the wound area was collected and RNA was extracted. The expression of M2 marker genes was analyzed by real-time RT- PCR. Data shown are the mean ratios ± SE of three separate experiments. P value: *<0.05. (TIF) [file pone.0076943.s004.tif]

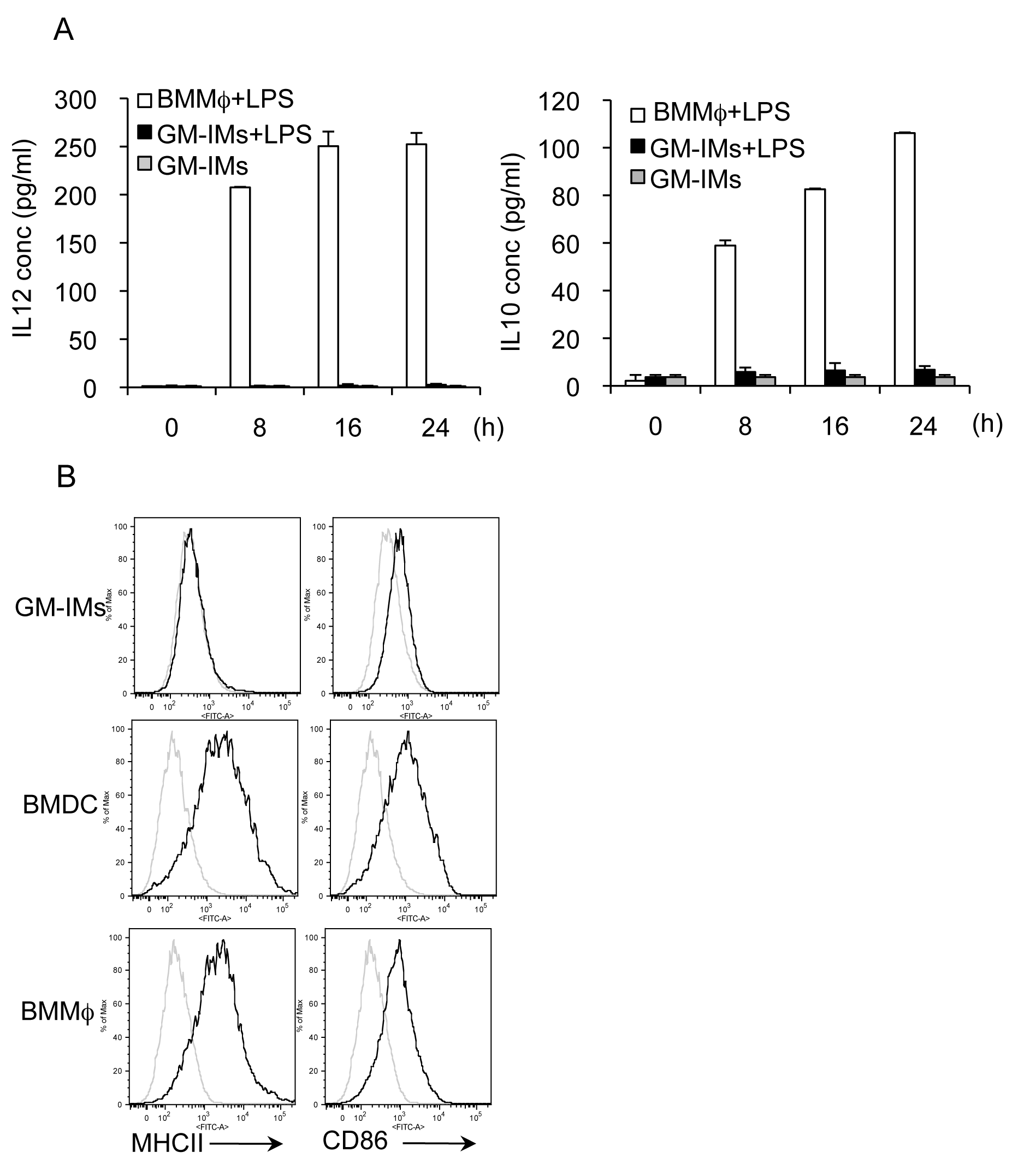

Supplement: Figure S5 — Markers of antigen presenting cells and regulatory macrophages. (A) GM-IMs or BMMφ (1 x 10^6^) were cultured in six-well plates with RPMI-1640 containing 10% FBS and 3% GM-CSF-CM. They were stimulated with one µg/mL LPS for indicated times. Supernatants were taken and expression of IL-12 or IL-10 was analyzed by ELISA. Data shown are the mean ratios ± SE of three independent experiments. (B) GM-IMs, BMDCs or BMM were stimulated with one µg/mL LPS. After 16 hr, they were stained with FITC-labeled anti-H-2d or FITC-labeled anti-CD86. They were analyzed by flow cytometry. Data are representative of three independent experiments. (TIF) [file pone.0076943.s005.tif]

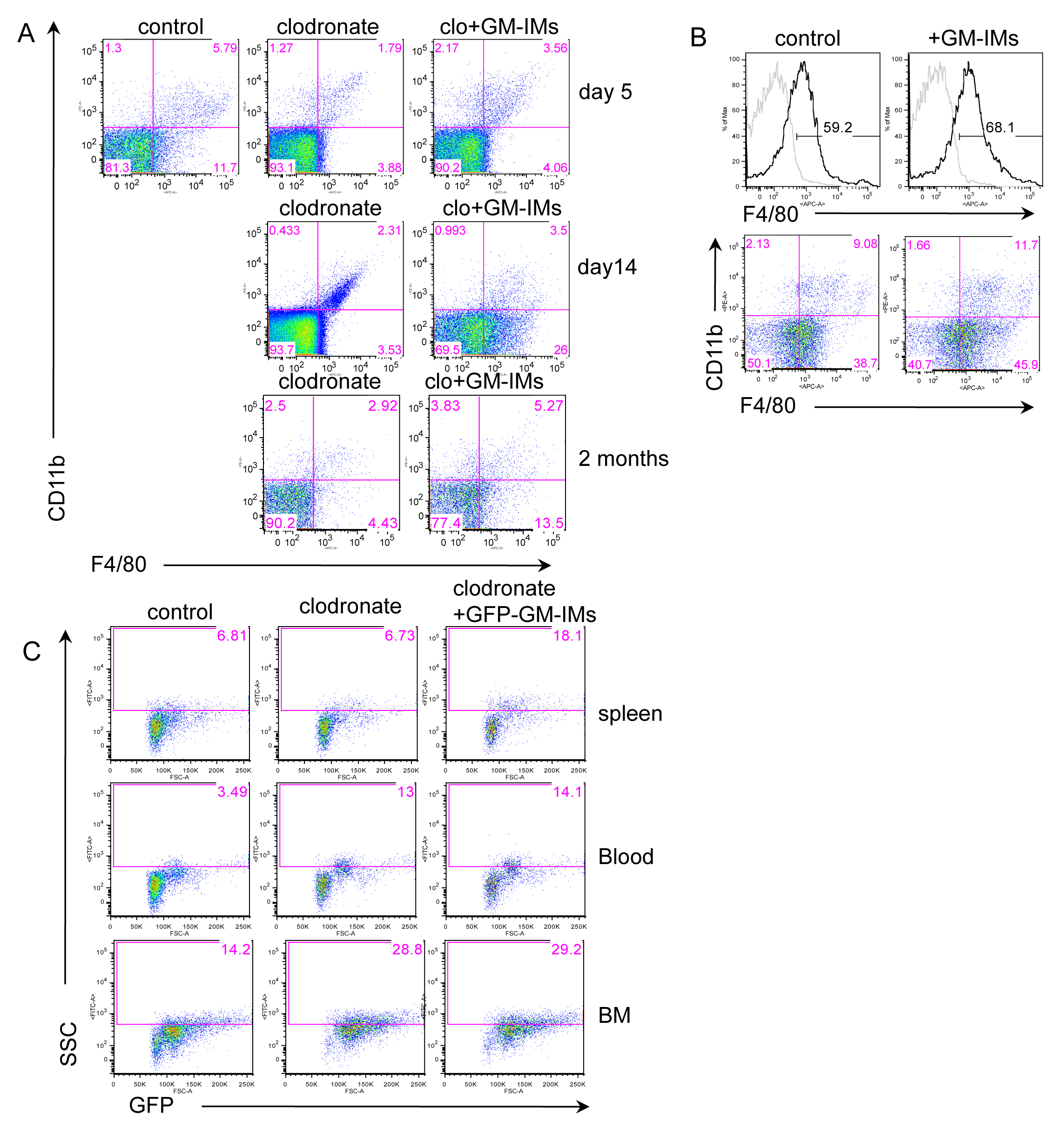

Supplement: Figure S6 — Injection of GM-IMs into macrophage-depleted mice. (A) Two-month-old female C57BL/6 mice were intravenously injected with clodronate liposomes. After two days, 5 x 10^6^ GM-IMs were injected intravenously. After five or 14 days or two months, spleen cells were stained with PE-labeled-anti-CD11b and APC-labeled-anti-F4/80 and analyzed by flow cytometry. (B) GM-IMs were injected into normal mice. At day 14, spleen cells were analyzed by flow cytometry. (C) GM-IMs from EGFP-C57BL/6 mice were injected intravenously after two days of clodronate injection. GFP-positive cells were analyzed by flow cytometry. The data was shown by dot-blot graph. Data are representative of three independent experiments (A-C). (TIF) [file pone.0076943.s006.tif]
