# Supplementary material for: Establishment of Self-Renewable GM-CSF-Dependent Immature Macrophages In Vitro from Murine Bone Marrow
Source: PLoS One. 2013 Oct 4;8(10):e76943. doi: 10.1371/journal.pone.0076943 (PMC3790761; doi:10.1371/journal.pone.0076943)
Supplement: Table S1 — Primer sequence used for RT-PCR. (DOCX) [file pone.0076943.s007.docx]

| Genes |  | Sequences (5'-3') |
| --- | --- | --- |
| *Irf5* | forward | GCTGGCTACAGGGTTCTGAG |
|  | reverse | CTGCTGGCTTCATTTCTTCC |
| *Socs3* | forward | GAGATTTCGCTTCGGGACTA |
|  | reverse | aacttgctgtgggtgaccat |
| *IL12b* | forward | AGGTCACACTGGACCAAAGG |
|  | reverse | TGGTTTGATGATGTCCCTGA |
| *IL6* | forward | ccggagaggagacttcacag |
|  | reverse | tccacgatttcccagagaac |
| *CCR2* | forward | AGAGAGCTGCAGCAAAAAGG |
|  | reverse | GGAAAGAGGCAGTTGCAAAG |
| *Nos2* | forward | AGGGAATCTTGGAGCGAGTTG |
|  | reverse | GCTTCAGGTTCCTGATCCAA |
| *Arg1* | forward | gtgaagaacccacggtctgt |
|  | reverse | ctggttgtcaggggagtgtt |
| *Retnla* | forward | tgctgggatgactgctactg |
|  | reverse | ctgggttctccacctcttca |
| *Mrc1* | forward | caaggaaggttggcatttgt |
|  | reverse | cctttcagtcctttgcaagc |
| *Chi3l3* | forward | gaaggagccactgaggtctg |
|  | reverse | cacggcacctcctaaattgt |
| *Csf1* | forward | TGTCCTGTCACAAGCTCTGG |
|  | reverse | GGCTTGCTTGGCTAGAGATG |
| *Csf2* | forward | atgcctgtcacgttgaatga |
|  | reverse | ccgtagaccctgctcgaata |
| *p21* | forward | cacagctcagtggactggaa |
|  | reverse | accctagacccacaatgcag |
| *Tert* | forward | AGCAAAAACCTTCCTCAGCA |
|  | reverse | AGTGAGCAGGCAGCTGGTAT |
| *Gapdh* | forward | aactttggcattgtggaagg |
|  | reverse | acacattgggggtaggaaca |
|  |  |  |

Supplemental Table 1
